# The psychological impact of quarantine on children with autism spectrum disorder

**DOI:** 10.1017/ipm.2020.117

**Published:** 2020-10-08

**Authors:** G. Byrne, E. N. Longphuirt

**Affiliations:** Psychology Department, Primary Care Services, HSE Community Healthcare East, Dublin, Ireland (Email: gary.byrne@hse.ie); Psychology Department, School Age Team, HSE Community Healthcare East, Dublin, Ireland

Given the imposition of unfamiliar health measures that infringe on personal freedoms, the closure of schools, the lack of contact from extended family members, and the exposure to COVID-19-related information from adults around them, it is perhaps not surprising that children are especially susceptible to psychological difficulties associated with the current pandemic (Danese *et al.*, 2020). Arising from these unprecedented times, a range of practical and useful guidelines have been offered by others in helping parents provide honest and clear information about the virus and the inherent uncertainty about the local and global effects of this (Dalton *et al.*, 2020). Research has highlighted the elevated levels of anxiety faced by children on the autism spectrum that is often exacerbated by changes in routine (White *et al.*, 2009). It could be reasonably expected that children on the autism spectrum may be particularly susceptible to the anxiety and uncertainty related to the societal changes brought about through the pandemic. A large number of families no doubt have to deal with the negative impact of quarantine on their children’s anxiety and it is imperative that such families with high levels of need still have remote access to service providers through this challenging time. However, within our own service in Dublin, we note that among a subsection of children, the opposite of what may be expected has occurred during the first 9 weeks of quarantine. Among this subgroup, parents reported that these children were calmer and that behavioural issues had for the most part decreased. These children would historically have come under the rubric of higher functioning or an Asperger’s type profile and the difficulties they face may be more nuanced than children with more complex developmental concerns. Parents have reflected that triggers such as morning routines were no longer an issue during quarantine. Similarly, children may no longer be faced with the many community triggers to their emotional and behavioural difficulties such as the performance anxiety associated with school or sense of rejection or failure often inherent in these contexts. Children may have also felt safe at home and in their family environments which are often sensitive to their needs. Parents have indicated that their other children had developed stronger bonds with their sibling with additional needs, becoming more inclined to help in daily routines. It may be that such enforced proximity, has engendered stronger bonds between siblings (Cluver *et al.*, 2020). In having time to attend more to the tasks of daily living parents also reported a fresh perspective on their child’s strengths. Certainly this perspective is an alternative to that provided when engaging in the prescribed educational curriculum. Unsurprisingly, recent reports of children’s well-being in the context of home schooling have indicated heightened levels of emotional and behavioural difficulties associated with school work time (Zhang *et al.*, 2020). It may be that some parents of children with additional needs had utilised the pandemic to focus on value-driven activities rather than those which fulfill societal expectations pre-pandemic. The inherently reinforcing value of activities such as baking, self-care, and leisure activities may have established a differential schedule of reinforcement for children, inadvertently weakening behaviours that challenge (O’Neill *et al.*, 1997). The above informal feedback from parents raises a number of questions as to how services implement strategies after quarantine has passed for this specific population. In addition, we recognise that although some children’s internalising and externalising difficulties may have reduced, parents may still be struggling with high levels of parental accommodation in helping reduce such behaviours (Feldman *et al.*, 2019). Similarly, the lack of social supports and isolation felt by many parents of children on the autism spectrum may be exacerbated during this unprecedented time (Byrne *et al.*, 2018).

Although a focus on the psychological health of children on the autism spectrum is important, mental health providers may also need to gauge how to help children re-engage with school and the community when restrictions are lifted. The likelihood of a gradual opening-up of society may pose difficulties for families as the incremental nature of same may restrict certain activities and supports. In addition, the psychological difficulties associated with the possible re-introduction of restrictions are factors that services must be mindful of. Schools may have a critical role in this in not only providing educational material but also offering children an opportunity to link in with familiar staff members. Services are well versed in helping children with autism transition between holidays and school terms but the uncertainty around school openings may hinder this. To the best of the authors’ knowledge no research to date has provided information about the psychological impact of quarantine on children on the autism spectrum. Recent reviews have reported on the significant psychological sequelae of quarantine (Brooks *et al.*, 2020). Further research is required in understanding how certain aspects of quarantine may in fact, on the surface help reduce anxiety amongst children with autism but also address the potential long-term impact that quarantine has on re-establishing important social routine and engagement.

## Disclaimer

The content of this article is the opinion of the authors and does not necessarily represent the official views of Health Service Executive.
